# The Impact of Treated Wastewater Irrigation on the Metabolism of Barley Grown in Arid and Semi-Arid Regions

**DOI:** 10.3390/ijerph19042345

**Published:** 2022-02-18

**Authors:** Alan Alvarez-Holguin, Gabriel Sosa-Perez, Omar Castor Ponce-Garcia, Carlos Rene Lara-Macias, Federico Villarreal-Guerrero, Carlos Gustavo Monzon-Burgos, Jesus Manuel Ochoa-Rivero

**Affiliations:** 1 Instituto Nacional de Investigaciones Forestales, Agricolas y Pecuarias (INIFAP), Campo Experimental La Campana, Aldama, Chihuahua 32910, Mexico; alvarez.alan@inifap.gob.mx (A.A.-H.); sosa.gabriel@inifap.gob.mx (G.S.-P.); ponce.omar@inifap.gob.mx (O.C.P.-G.); lara.rene@inifap.gob.mx (C.R.L.-M.); 2 College of Animal Production and Ecology, Autonomous University of Chihuahua, Chihuahua 31453, Mexico; fvillarreal@uach.mx (F.V.-G.); carlosmonzon19@gmail.com (C.G.M.-B.)

**Keywords:** agriculture, nutritional composition, photosynthetic efficiency, stomata, wastewater reuse

## Abstract

The use of treated wastewater (TWW) for irrigation has gained global attention since it reduces pressure on groundwater (GW) and surface water. This study aimed to evaluate the effect of TWW on agronomic, photosynthetic, stomatal, and nutritional characteristics of barley plants. The experiment with barley was established on two bands: one band was irrigated with GW and the other with TWW. The evaluation was performed 25, 40, 60, 90, and 115 days after sowing (DAS). Results showed that irrigation with TWW increased (*p* < 0.01) grain yield by 54.3% and forage yield by 39.4% compared to GW irrigation. In addition, it increased plant height (PH) (*p* = 0.013), chlorophyll concentration index (CCI) (*p* = 0.006), and leaf area index (LAI) (*p* = 0.002). TWW also produced a positive effect (*p* < 0.05) in all the photosynthetic efficiency parameters evaluated. Barley plants irrigated with TWW had lower stomatal density (SD) and area (SA) (*p* < 0.001) than plants irrigated with GW. Plants irrigated with TWW had a higher P concentration (*p* < 0.05) in stems and roots and K concentration in leaves than plants irrigated with GW. We concluded that the use of TWW induced important biochemical, physiological, and agronomic changes in barley plants. Hence, the use of TWW may be a sustainable alternative for barley production in arid and semi-arid regions. This study was part of a government project, which aimed to develop a new metropolitan irrigation district with TWW. This study may contribute to the sustainability of water resources and agricultural practices in northern Mexico.

## 1. Introduction

Anthropic activities, population growth, and climate change have caused a decrease in water quality and quantity, generating huge amounts of wastewater (WW). Around 380 billion m^3^ of WW are generated globally each year, and this amount could increase 24% by 2030 and 51% by 2050 [1]. Therefore, there is a need of alternatives for the use of WW on a sustainable basis.

Agriculture is the activity with the highest water demand on Earth, and this demand is exacerbated in arid and semi-arid lands, where water availability is limited [2]. According to the FAO, 2.75 million km^2^ are irrigated with WW around the world [3]; however, more than 80% of WW produced worldwide is released into the environment without any treatment. Thus, the use of treated wastewater (TWW) for agricultural irrigation has gained global attention since water consumption has significantly increased due to the growing demand for food [4]. Currently, around 20 million hectares are irrigated with TWW in 50 countries, contributing to 40% of the food produced [5].

The use of TWW in agriculture can generate numerous benefits. For instance, the use of TWW for agricultural irrigation reduces pressure on groundwater (GW) and surface water, which could be directed towards domestic use [6,7,8]. Moreover, the use of TWW is cheaper than pumping GW [9,10].

From an ecological perspective, irrigation with TWW decreases the eutrophication rate of aquatic ecosystems [1]. It has been shown that TWW can considerably increase the concentration of macro (N, P, and K) and micronutrients (Fe, Mn, Zn, and Cu), which increases soil fertility [11,12]. Qadir et al. [1] reported that worldwide, TWW carries 16.6 Tg (Tg, million metric ton) of N, 3.0 Tg of K, and 6.3 Tg of P annually. This amount represents 13.4% of the global demand for nutrients in agriculture. In addition, the increase in nutrient concentration decreases fertilization needs and reduces costs [13]. Previous research has found that TWW decreases the use of fertilizers from 50% to 100% in forage species, such as alfalfa and wheat [14,15]. In this sense, the use of TWW contributes to reduce the use of agrochemicals, generating positive ecological effects [1,7]. Hence, there is a great opportunity for using TWW in agriculture.

Several studies have revealed that the use of TWW can improve physicochemical properties and enhance the productivity of many economically important crops [5,16,17]. For example, the use of TWW enhanced the chlorophyll content, photosynthetic efficiency, and forage yield in alfalfa (*Medicago sativa*) [18]. Likewise, the irrigation with TWW improved the forage yield of kikuyu grass (*Pennisetum clandestinum*) in the absence of chemical fertilizers compared to the irrigation with GW [19]. Furthermore, the use of this source of water increased the chlorophyll fluorescence, stomatal conductance, and the photosynthetic rate and decreased stress metabolites, such as leaf phenolic compounds and carbohydrates in olive trees (*Olea europaea*) [20]. Such benefits were induced by the high concentration of nutrients in the TWW.

Previous studies have shown that TWW irrigation also has beneficial effects on barley (*Hordeum vulgare* L.) productivity [14,21,22]. This crop is the fourth most important cereal in the world, just behind wheat, corn, and rice [21]. Around 150 million tons year^−1^ of barley are globally produced. In 2013, barley exports and imports were valued at USD 8.5 and USD 9.4 trillion, respectively [22]. However, studies on the physiological and biochemical effects on this species due to TWW irrigation are limited. Hence, the objective of this study was to evaluate the effect of TWW on agronomic, photosynthetic, stomatal, and nutritional characteristics of barley plants. This information will build a deeper understanding of the use of TWW in agriculture. It may also contribute to reducing the city’s freshwater requirements. Thus, it may contribute to the sustainability of water resources and agricultural practices, consequently improving the availability of this natural resource.

## 2. Materials and Methods

### 2.1. Study Area and Experimental Setup

**Figure 1:**
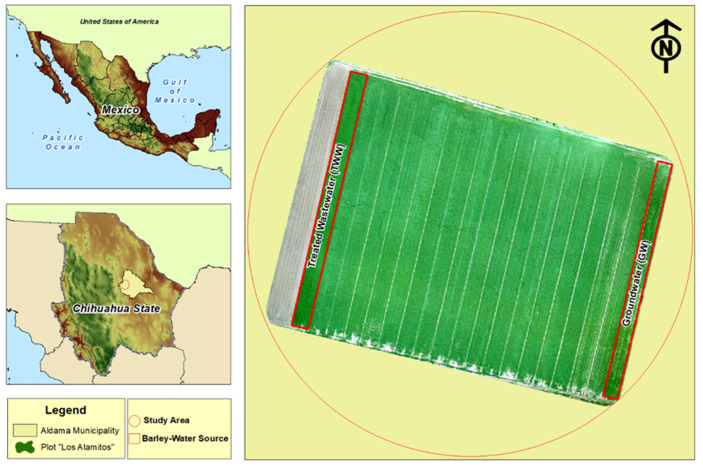
Location of the plot under study (municipality of Aldama) and an aerial image showing its characteristics.

The experiment was conducted during 2020 on a plot identified as “Los Alamitos”, located in the municipality of Aldama, in the state of Chihuahua, Mexico (Figure 1). The plot is located at 28°44′44.28″ N and 105°57′28.52″ W. The climate is dry semi-warm, with a mean maximum temperature of 23.5 °C during summer and a mean minimum temperature of 5.8 °C during winter. The mean annual precipitation is 298 mm, which mainly occurs during July–September [23,24]. The plot under study had 19 stripes or bands 10-m wide and 150-m long, with aisles of 1.0 m in between bands (Figure 1). These bands have conventionally been irrigated for 30 years with two water sources: one section of three bands with GW and the rest with TWW. The experiment with barley was established on two bands: one band was irrigated with GW and the other with TWW. The distance between these two experimental bands was 150 m. The remaining bands were sown with oats. The TWW used for irrigation was from a wastewater treatment plant (WWTP) located in the south of Chihuahua City. The WWTP has a secondary treatment with an effluent of 1100 L seg^−1^ from domestic, industrial, and storm sewages.

The soil preparation for sowing was carried out from 10–25 January by performing the following practices: fallow, tracing, subsoiling, and furrowing. The soil characteristics were determined before sowing by analyzing a composite soil sample, which was extracted from five subsamples collected in the upper soil profile (0–30 cm). The composite sample was analyzed according to Mexican Standard NOM-021-RECNAT-2000 at the National Laboratory of Water, Soil, Plant, and Atmosphere of INIFAP, located in Gomez Palacio, Durango, Mexico. The physicochemical characteristics of the soil under study can be seen in Table 1.

The experimental design consisted of randomized blocks. The band of each treatment or water source was divided into six blocks 25-m long. Seeds of barley, var. “Alina”, were sown on 1 February 2020, using a seeding rate of 120 kg ha^−1^. Five days after sowing (DAS), 100 kg ha^−1^ of urea (CO(NH_2_)_2_) were applied to the soil surface by using a tractor-driven fertilizing machine. Furrow irrigation events were carried out on 7 February, 21 February, 6 March, 23 March, 3 April, and 17 April, with 120 mm per irrigation. To dismiss differences in the results due to high soil moisture content in some of the treatments, the soil moisture (volumetric water content) was measured with a FieldScout TDR 300 portable meter (Time-Domain Reflectometer- Spectrum Technologies Inc., Aurora, IL, USA) one or two days before each watering, beginning in March. The moisture was measured using 20-cm rods at randomly selected points on one band irrigated with TWW and one band with GW. Soil moisture was similar between the treatments, and the general mean of the volumetric water content, before each irrigation date, was 18.7% (s.d. = 0.9%), 19.3% (s.d. = 1.3%), 20.9% (s.d. = 1.6%), and 21.7% (s.d. = 2.6%), respectively.

Water quality was analyzed for each water source (GW and TWW). For that, two water samples from each water source were collected at each irrigation event, beginning on 21 February. A total of 10 water samples was collected from each source by the time the experiment concluded. The on-site measurements included pH, electrical conductivity (EC), and temperature. Concentrations of nitrates (NO_3_^−^ as N), phosphates (PO_4_^3−^), and sulfates (SO_4_^2−^) were analyzed at the College of Chemical Sciences of the Autonomous University of Chihuahua. The concentrations of heavy metals and metalloids were determined at the Mexican Geological Service (SGM), located in Chihuahua City. The analyses were based on the Mexican Regulation NOM-001-SEMARNAT-1996 and [25]. The results are presented in Table 2. In general, the values of the water quality parameters from the two sources were within acceptable levels according to the Mexican Regulations for irrigation [26].

### 2.2. Agronomic Attributes

Plant height (PH), chlorophyll concentration index (CCI), and leaf area index (LAI) were evaluated 25, 40, 60, and 90 DAS. PH was measured on three randomly selected plants from each block. Measurements of CCI (CCM-200 device, Opti-Sciences, Inc., Hudson, NH, USA) were made on the second leaf of three plants from each block. Likewise, measurements of LAI were performed with a ceptometer (LP-80, Decagon Devices, Inc., Pullman, WA, USA) in areas of 0.25 m^2^ (0.4 × 0.62 m), with one sample per block (*n* = 6). The sensor bar of the ceptometer was positioned at 0.05 m above the ground level, and two measurements were taken every 0.1 m. Meanwhile, the external PAR (photosynthetically active radiation) sensor of the ceptometer was positioned at 1.5-m height, at an angle of 90° with respect to the ground level, in a shadow-free location.

Forage yield was also determined on the aforementioned sampling dates. For that, forage cuts were made at 0.05 m from the ground level in six quadrants (*n* = 6) of 0.25 m^2^ per treatment or water source. The samples were then stored in paper bags and dried at 65 °C during 72 h. An additional sampling was performed at 115 DAS to calculate grain yield. For that, the seed was separated from the forage and weighed to estimate grain yield.

### 2.3. Photosynthetic Efficiency

The photosynthetic efficiency was evaluated through the fluorescence parameters of chlorophylls: maximum quantum yield of photosystem II (Fv/Fm), photochemical efficiency of photosystem II (ΦPSII), quantum efficiency of unregulated energy dissipation in PSII (YNO), quantum efficiency of regulated energy dissipation in the PSII (YNPQ), and electron transfer rate (ETR). These parameters were evaluated using a portable photosynthesis yield analyzer (Mini-PAM II; Walz, Effeltrich, Germany).

Photosynthetic efficiency was evaluated 80 DAS, when the plants were at their flowering stage. This evaluation was performed in three plants per block (i.e., 18 plants per treatment) and consisted of randomly measuring three healthy leaves from each plant. The measurements were done on both dark-acclimated (one hour after dark) and light-exposed (11:30 a.m.–12:30 p.m.) leaves. At night, the plants were subjected to a photon light pulse of approximately 0.5 µmol m^−2^ s^−1^, with a frequency of 600 Hz, to define the minimum fluorescence signal (F0). A photon saturation pulse of approximately 6000 µmol m^−2^ s^−1^ was then applied during 0.8 s to find the maximum fluorescence signal (Fm). The parameters of F0 and Fm served to calculate the maximum quantum yield of photosystem II through the equation proposed by Kitajima and Butler [28]:

Fv/Fm = (Fm − F0)/Fm

These steps were repeated at noon to find the fluorescence level (Ft) and the maximum fluorescence level (F’m) of light-exposed leaves.

The values obtained from the aforementioned measurements were used to calculate the photochemical efficiency of photosystem II by using the equation proposed by Genty et al. [29]:

ΦPSII = (F’m − Ft)/F’m

In addition, this equation was used to calculate the quantum efficiency of unregulated energy dissipation in the PSII:

YNO = Ft/Fm

The quantum efficiency of the regulated dissipation of energy in the PSII was obtained by the following equation:

YNPQ = (Ft/F’m) − (Ft/Fm).

Finally, the ETR was calculated using this equation:

ETR = [(ΦPSII) × (PAR radiation received) × (0.84)]

Data were collected and processed in the WinControl-3 software, version 3.23.

### 2.4. Stomatal Characteristics

Stomata of experimental plants were characterized by assessing stomatal density (SD) and stomatal area (SA). For that, leaf blade imprints were extracted from 18 plants per treatment, three plants per block, and three healthy leaves per plant. Imprints were taken from approximately 1 cm^2^ of adaxial and abaxial leaf surfaces. The imprints were then analyzed under a phase contrast microscope (Model Axio imager 2, Carl Zeiss, Jena, Germany) at a magnification of 200×. Five fields of view per imprint were photographed with an AxioCam MRc5 camera (Carl Zeiss). The number of stomata was counted, and data were normalized to 1 mm^2^. The SA was calculated from three randomly selected stomata per optic field by using the Zen 2 core software. A total of 810 stomata per treatment were analyzed from 270 optical fields.

### 2.5. Analysis of Nutritional Components

The nutritional components of the plants were analyzed at the National Laboratory of Water, Soil, Plant, and Atmosphere of INIFAP located in Durango, Mexico. A total of 24 plants (12 plants per treatment) were randomly selected and split into roots, stems, leaves, and grain. The samples were dried and homogenized. Then, nitrogen (N), phosphorus (P), potassium (K), calcium (Ca), magnesium (Mg), sodium (Na), and iron (Fe) were quantified using acid digestion of samples with 0.5 g of plant tissue. The digested samples were analyzed in the atomic absorption equipment AAnalyst700-Perkin Elmer. All the analyses were determined according to standard methods [30,31,32].

### 2.6. Statistical Analysis

Data were analyzed using a multi-factor analysis of variance. The variables of PH, CCI, and LAI were analyzed using a four-factor model utilizing water source, sampling date, block, and plant as factors. The plant was included in the analysis to avoid pseudo-replication. The forage yield analysis was adjusted to a three-factor model (water source, sampling date, and block) since it was evaluated by area and not by plants. Grain yield, photosynthetic parameters, stomatal characteristics, and nutritional components were analyzed from plants of a single sampling date; thus, data were analyzed using three factors; water source, block, and plant. Regarding the physicochemical characteristics, water from the two sources, from each sampling date, were compared using Dunnett’s comparison test (α = 0.05). Data of soil moisture were analyzed using repeated measurements with two factors (water source and watering date), where the subjects were the bands. Data were analyzed using the R software version 4.0.5.

## 3. Results and Discussion

### 3.1. Treated Wastewater Improve Agronomic Traits on Barley

**Figure 2:**
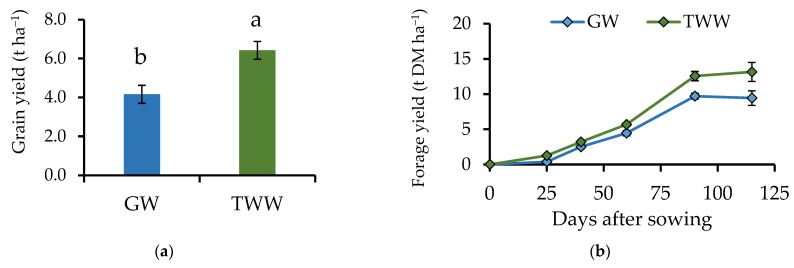
(**a**) Grain and forage yield in barley plants (*Hordeum vulgare*) irrigated with treated wastewater (TWW) and groundwater (GW). (**b**) Forage yield was evaluated at 25, 40, 60, and 90 days after sowing (DAS). Grain yield was evaluated 115 DAS. *n* = 6; *p* = 0.007 for grain yield and *p* = 0.006 for forage yield. Different letters indicate significant differences between water sources (Dunnett test; *p* < 0.05) and black bars represent the standard error.

Irrigation with TWW increased grain yield by 54.3% (*p* = 0.007) compared to GW irrigation (Figure 2a). Samarah et al. [21] found similar results in different barley cultivars, where the use of TWW increased grain yield. The increase in grain yield is attributed to the enriched mix of nutrients contained in the TWW, which benefits crops’ growth and productivity [1,33,34].

Grain yield with TWW was 6.42 t ha^−1^, using 46 N units (100 kg ha^−1^ of urea). Ramírez-Novoa et al. [35] obtained a yield of 7.36 t ha^−1^ of the same variety of barley “Alina”; however, the fertilizer applied in that study was 45-60-00 (urea and triple calcium superphosphate) at the time of sowing and 45-00-00 during the first irrigation. The results prove the feasibility of producing barley with low fertilization costs using TWW. This is also supported by other studies, where the nutritional contribution of TWW to crops was reported [1].

**Figure 3:**
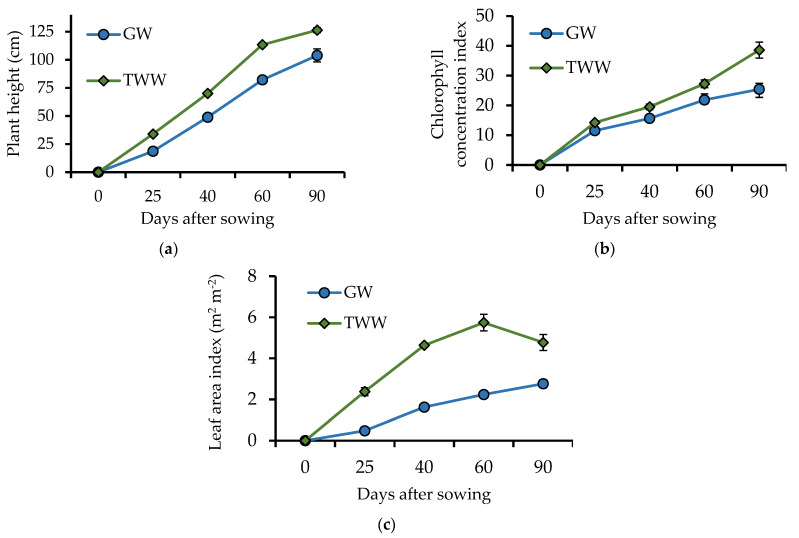
(**a**) Plant height, (**b**) chlorophyll concentration index, and (**c**) leaf area index of barley plants (*Hordeum vulgare*) irrigated with treated wastewater (TWW) and groundwater (GW). Sampling days were 25, 40, 60, and 90 days after sowing. *n* = 6; *p* = 0.013 for plant height, *p* = 0.006 for chlorophyll concentration index, and *p* = 0.002 for leaf area index. The black bars represent the standard error.

The maximum forage yield using TWW was 39.4% higher (*p* = 0.006) than using GW, and the difference was consistent during all the evaluations (Figure 2b). Previous studies have also found that irrigation with TWW can generate an increase in forage yield [33]. For instance, Elfanssi et al. [18] found that irrigating with TWW increased productivity in alfalfa (*Medicago sativa*), and Aghtape et al. [34] reported an increase in yield and forage quality in foxtail millet (*Setaria italica*) when irrigated with TWW. In this study, forage yield, PH (*p* = 0.013), CCI (*p* = 0.006), and LAI (*p* = 0.002) were higher using TWW instead of GW (Figure 3). These findings are explained by the chlorophyll since it is the pigment responsible for capturing solar radiation for photosynthesis, and it occurs mainly on the leaves. Tambussi et al. [36] found that barley cultivars with a larger leaf area are more efficient in terms of photosynthesis and produce a greater amount of forage and grain.

### 3.2. Treated Wastewater Enhanced the Photosynthetic Efficiency of Barley Plants

**Figure 4:**
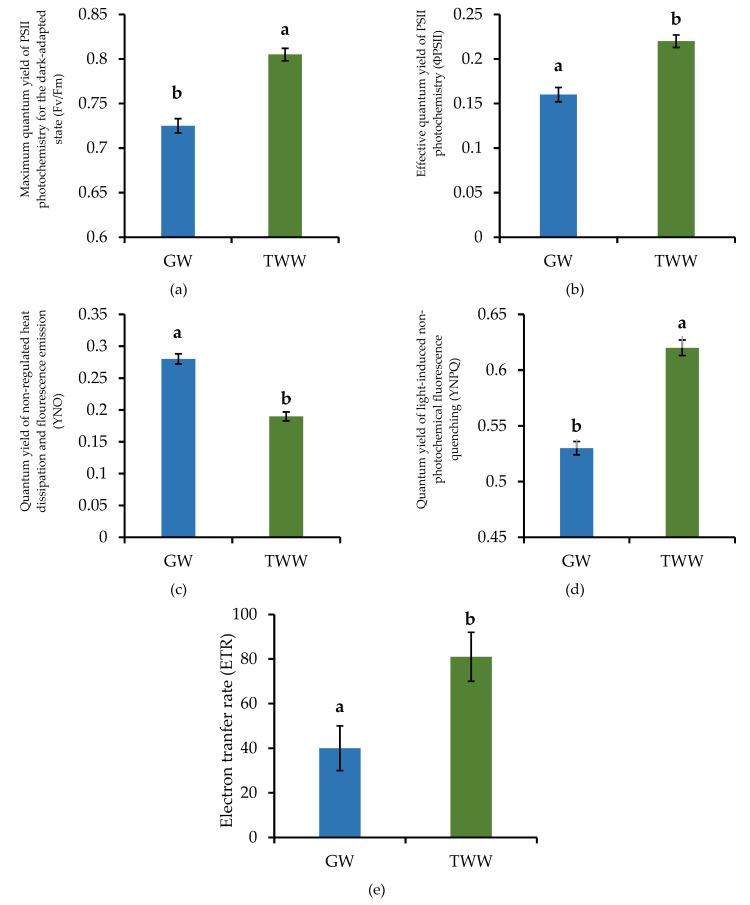
Photosynthetic efficiency parameters of barley plants (*Hordeum vulgare*) irrigated with groundwater (GW) and treated wastewater (TWW). *n* = 18; *p* < 0.001 for Fv/Fm (**a**), *p* = 0.011 for ΦPSII (**b**), *p* = 0.004 for YNO (**c**), *p* = 0.004 for YNPQ (**d**), and *p* = 0.044 for ETR (**e**). Different letters indicate significant differences between water sources (Dunnett test; *p <* 0.05) and black bars represent the standard error.

The water source for irrigation significantly influenced (*p* < 0.05) all the photosynthetic efficiency parameters evaluated (Figure 4). Plants irrigated with TWW presented higher (*p* < 0.001) maximum photochemical efficiency (Fv/Fm) than those irrigated with GW. This difference was consistent with the highest CCI found in the plants irrigated with TWW. These results agree with those reported by Palliotti et al. [37], who found that high concentrations of chlorophyll benefit the absorption of light and increase Fv/Fm. Chlorophyll is the pigment responsible for capturing solar radiation for photosynthesis; therefore, it is correlated with Fv/Fm [38,39].

The Fv/Fm for plants irrigated with GW was 0.72. This was lower than the 0.80 found in plants irrigated with TWW. These results suggest plants with GW were under stress since values of Fv/Fm lower than 0.80 indicate damage in the photosynthetic apparatus. All the factors causing inhibition of the reaction centers of PSII increase energy dissipation [40,41,42,43]. In the same way, the plants irrigated with GW had higher Y(NO) (*p* = 0.004) than the plants irrigated with TWW. The Y(NO) index measures the amount of non-regulated energy dissipated, which is a detrimental form of dissipation [44]. These results are consistent with several studies reporting that plants under stress have lower Fv/Fm and higher Y(NO) compared to healthy plants. For example, Shu et al. [45] found that salinity decreased Fv/Fm and increased Y(NO) in cucumber plants (*Cucumis sativus*). Similarly, Marriboina and Attipalli [46] found the same effect in Indian bean plants (*Pongamia pinnata*) under salinity stress, while Song et al. [47] reported a significant decrease in Fv/Fm in rice plants (*Oryza sativa*) caused by heat stress.

The plants with TWW lost more energy due to heat dissipation, as estimated by Y(NPQ), which is an indicator of regulated energy dissipation and is associated with the xanthophyll cycle and acidification of the thylakoid lumen. Xanthophylls are three carotenoids (violaxanthin, anteroxanthin, and zeaxanthin) involved in heat dissipation [48]. Under radiation stress conditions, violaxanthin is converted to zeaxanthin by the enzyme violaxanthin de-epoxidase. This set of reactions is known as the xanthophyll cycle. The binding of protons and zeaxanthin light-collecting antenna proteins in thylakoids causes conformational changes leading to energy capture and heat dissipation [49]. The violaxanthin de-epoxidase enzyme is located in the lumen of the thylakoids and is activated at acidic pH [50]. For this reason, the acidification of the thylakoid lumen is also involved in the dissipation of excess light energy in the form of heat.

Stress from excess radiation can induce an increase in Y(NPQ) [51]. Plants under stress conditions due to radiation can be exposed to excess energy, which can damage the PSII if the energy is not dissipated in a regulated manner [52]. High temperatures therefore can affect the thylakoid membrane and disrupt the electron donor and acceptor complexes in PSII [53,54]. In addition, excess of undissipated energy can react with molecular oxygen and create free radicals, which damage the photosynthetic apparatus [55]. Then, heat stress commonly causes a significant reduction in Fv/Fm and ΦPSII [47]. Overall, the results suggest that plants irrigated with TWW dissipated excess heat in a better way since they obtained higher Y(NPQ) and lower Y(NO) than plants irrigated with GW.

The plants irrigated with TWW also had higher ΦPSII (*p* = 0.011) and ETR (*p* = 0.044) than plants irrigated with GW, suggesting that those plants transform solar radiation into energy molecules (i.e., ATP) more efficiently. Results of the photosynthetic parameters are consistent with the grain and forage yield since plants irrigated with TWW had the highest values. Tambussi et al. [36] reported that barley cultivars with the highest ΦPSII were the most productive.

### 3.3. Treated Wastewater Produced Changes in Stomatal Density and Area of Barley Plants

**Figure 5:**
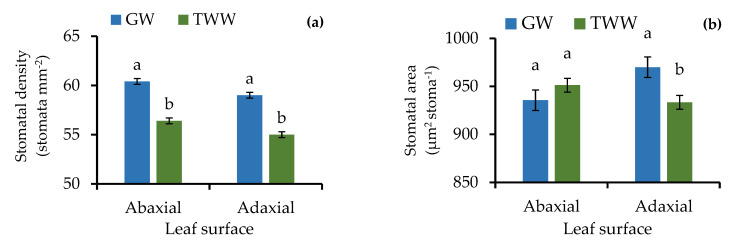
(**a**) Stomatal density and (**b**) stomatal area of barley (*Hordeum vulgare*) irrigated with groundwater (GW) and treated wastewater (TWW). *n* = 18; *p* < 0.001 for abaxial and adaxial stomatal density, *p* > 0.05 for abaxial stomatal area, and *p* < 0.001 for adaxial stomatal area. Different letters indicate significant differences between water sources (Dunnett test; *p <* 0.05) and black bars represent the standard error.

**Figure 6:**
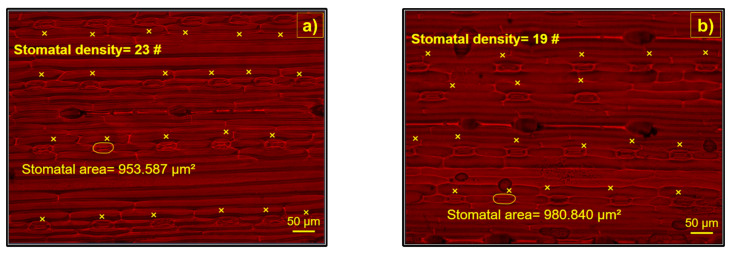
Microstructure of the abaxial leaf surface of barley plants irrigated with (**a**) treated wastewater (TWW) and (**b**) groundwater (GW). “#” = number of stomata per field of view. “×” = stomata accounted.

The water source for irrigation (GW and TWW) produced different SDs and SAs between the treatments (Figure 5 and Figure 6). The barley plants irrigated with TWW had lower SD than the plants irrigated with GW (*p* < 0.001), both on the adaxial and abaxial leaf surfaces. In contrast, the SA was similar on the abaxial leaf surfaces (*p* > 0.05) but different on the adaxial surface, with the lowest SA in the plants irrigated with TWW (*p* < 0.001).

Stomatal characteristics are important for the plant’s physiology since these pores control the loss of water through transpiration and the CO_2_ assimilation through photosynthesis [56,57]. The stomatal size and density, therefore, have been important for genotype selection and plant breeding research [58]. For instance, Franks et al. [59] found that the reduction of SD, through overexpression of the EPF2 gene, decreased the stomatal conductance and increased the water-use efficiency in mutant *Arabidopsis* lines. Likewise, Li et al. [60] detected that wheat (*Triticum aestivum*) cultivars with low density and SA showed less transpiration and increased the photosynthetic rate and water-use efficiency. The reason could be that smaller stomata can open and close faster, increasing CO_2_ assimilation and decreasing transpiration [61,62,63]. Hughes et al. [64] also reported that mutant barley lines with low SD showed higher ΦPSII, resulting in better water-use efficiency under stress conditions. The results of the present study then suggest that the high photosynthetic efficiency found in plants irrigated with TWW was related to the low density and SA and have a positive effect on forage and grain yields.

### 3.4. Treated Wastewater Changed the Nutritional Composition of Barley Plants

Regarding the concentration of nutrients in plants, N had the highest (*p* < 0.05) in leaves and grains of plants irrigated with GW (Table 3). These results are consistent with those from the water analyses, as the concentration of NO_3_^−^ (1.78 mg L^−1^) was higher in plants irrigated with GW than in plants irrigated with TWW (Table 1). The high content of NO_3_^−^ in GW is an indicator of contamination and may be the result of poor management of agricultural plots, especially related to N fertilizers [65,66], and could be also the effect of irrigation with TWW [8].

Since N is a fundamental part of chlorophyll molecules, it was expected that the high concentration of N in GW plants would lead to a high CCI; however, this index was higher in plants irrigated with TWW. This could be explained by the molybdenum (Mo) concentration as follows: plants absorb N either in the form of ammonium (NH_4_^+^) or NO_3_^−^. For N-NO_3_^−^ to be assimilated, it must be reduced to NH_4_^+^ through the action of the enzymes NO_3_^−^ and NO_2_^−^ reductase [67]. These enzymes require Mo as an enzymatic cofactor, and the absence of this element could cause low assimilation of N-NO_3_^−^. In the absence of Mo, plants fertilized with NO_3_^−^ present a poor growth and low concentrations of chlorophyll and ascorbic acid and a high content of NO_3_^−^ [68]. The concentration of Mo was 0.01 mg L^−1^ higher in the TWW plants (*p* < 0.05) than in the GW plants (Table 1), and this may explain the high CCI and photosynthetic efficiency of plants irrigated with TWW. Meanwhile, the plants irrigated with GW may have had assimilated less N due to lower concentrations of Mo in the plants’ tissue.

Nonetheless, the characteristics of GW plants could be of interest to the beer industry. The N content of the grain influences the quality of malt production because it is related to nitrogenous compounds, such as proteins, amino acids, amines, and purines. For this reason, the N content significantly influences beer production, as it is important for yeast fermentation [69]. In addition, it is related to beer quality parameters, especially color, texture, turbidity, foam formation, CO_2_ retention, and microbial nutrition [70]. Then, the grain produced with GW may have a higher malting quality than that generated with TWW since it presented a higher N concentration. Regarding P, the stem and root tissues of the plants irrigated with TWW presented the highest concentration of this nutrient (*p* < 0.05). In contrast, the stem, root, and leaf tissues of the plants irrigated with GW had the highest concentration of Mg (*p* < 0.05). These results are consistent with the nutrient concentrations found in the water since the TWW plants had the lowest concentration of PO_4_^3−^ (6.80 mg L^−1^; Table 2) and the highest concentration of Mg in soil (2.07 meq 100 g^−1^). The high content of P-PO_4_^3−^ in TWW may be a result of the degradation of organic materials contained in these water sources, as it has been reported in previous research [71,72]. Conversely, the high Mg content has been more related to the type of rock in the subsoil [66,73].

Plants irrigated with TWW had a higher concentration of P in stems and roots (*p* < 0.05) than the GW plants, which is consistent with the high concentration of P found in these water sources [74,75]. Previous research has found a strong relationship between chlorophyll concentration and P content in various crops [76,77,78,79,80], which suggests that the biosynthesis of chlorophyll molecules depends partially on the assimilation of P. These results are consistent with the ones from the present study since the CCI was higher in the plants irrigated with TWW. Furthermore, the higher concentration of P in stems of TWW plants could be partially explained by the higher photosynthetic efficiency since the P concentration is related to different photosynthetic parameters, such as Fv/Fm and ΦPSII [77,81]. The concentration of P in leaves was similar between the two treatments (*p* > 0.05). P is a highly mobile element within the plant [82]; thus, it could have been in the leaves at the time of measurement of photosynthetic parameters. The photosynthetic parameters were evaluated during the flowering stage, while the nutritional evaluation was evaluated on mature plants.

The K concentration in leaves was higher in plants irrigated with TWW (*p* < 0.05). The nutrient K controls the entry and exit of water to the cells and therefore the opening and closing of the stomata [83,84]. Hence, the high concentration of K in plants irrigated with TWW could have influenced the differences found in SD and SA. Previous research has reported a relationship between K and stomata. For instance, Shabala et al. [85] found that a mechanism to tolerate salinity is to increase the concentration of K in the leaves and decrease SD in quinoa plants (*Chenopodium quinoa*). Benlloch-González et al. [86] reported that K deficiency inhibited stomatal closure, which induced a state of water stress and affected growth in olive trees (*Olea europaea*) and sunflower plants (*Helianthus annuus*). In contrast, the high concentration of K in plants irrigated with TWW may have also contributed to produce the differences found in grain yield, forage yield, LAI, and CCI. Accordingly, Zhang et al. [87] reported a significant interaction between K, LAI, and CCI in potatoes (*Solanum tuberosum*). According to the authors, a high concentration of K promoted high tuber yield and quality, which agrees with our findings. It is important to point out that the N, P, and K concentrations in leaves were below the ranges indicated by Havlin et al. [88] for barley. In this sense, Jones [82] mentioned that N, P, and K are elements of high mobility in the plant, and as the age of the crop advances, the concentration of these nutrients decreases. The sampling in the present study was carried out during harvest, and this may explain the low concentration of these nutrients. However, the concentration of nutrients in the grains was higher than the values found in previous research. For instance, Dung et al. [89] reported values of 4900, 1000, and 2200 mg Kg^−1^ for K, Mg, and P, respectively. Similar results are reported by the USDA with 2800, 790, and 2210 mg Kg^−1^ of K, Mg, and P in the grain, respectively. The high concentration of nutrients in the grain and the low concentration in the leaf then suggest that the nutrients (K, Mg, and P) were moved from the leaves to the grains at the time of the measurements.

## 4. Conclusions

TWW is an important source of nutrients since it induces important agronomic, photosynthetic, stomatal, and nutritional changes in barley plants. Plants irrigated with TWW showed higher grain yield, forage yield, PH, CCI, and LAI. The photosynthetic efficiency increased in plants irrigated with TWW. Furthermore, plants irrigated with TWW had lower SD and SA than GW plants. That may be a mechanism of adaptation to the stress generated by the increase in biomass and leaf area. Agronomic, biochemical, and physiological attributes in plants irrigated with TWW appear to be linked to better assimilation of K and P. Overall, the use of TWW represent a sustainable alternative for barley production in arid and semi-arid regions. Nevertheless, the evaluation of concentrations of toxic elements, emergent pollutants, and microplastics in the plant, water, and soil after using TWW is highly recommended.

This study may contribute to the sustainability of water resources and agricultural practices in northern Mexico since it was part of a government project that aimed to develop a new metropolitan irrigation district.

## Figures and Tables

**Table 1:** Physicochemical characteristics of the soils irrigated with groundwater (GW) and treated wastewater (TWW).

Variable	Units	GW	TWW
pH		8.51	8.44
Electrical Conductivity (EC)	dS m^−1^	1.17	1.01
Texture		Clay loam	Clay loam
Cation Exchange Capacity (CEC)	meq 100 g^−1^	36.01	32.71
* Organic matter (OM)	%	1.3	1.5
Nitrogen (N-NO_3_−)	mg kg^−1^	29.11	9.49
^1^ Phosphosrus available (P)		8.24	12.76
^2^ Removable potassium (K)		693.98	895.39
^3^ Removable calcium (Ca)		4102.53	4702.02
^4^ Removable magnesium (Mg)		449.26	388.48
* Copper (Cu)		1.59	1.20
* Iron (Fe)		0.6	1.76
* Manganese (Mn)		2.66	2.30
* Zinc (Zn)		2.01	3.31

^1,2,3^ Units are kg ha^−1^ and reported as P_2_O_5_, K_2_O, and CaO, respectively; ^4^ reported MgO; * units are mg Kg^−1^.

**Table 2:** Physicochemical characteristics of the water used for the irrigation of barley (*Hordeum vulgare)*.

		GW			TWW			
Variable	Units	Mean	Min	Max	Mean	Min	Max	Quality Standards
pH		7.67	7.18	8.89	7.78	7.25	8.75	6.0–9.0 *
Electrical Conductivity (EC)	mS cm^−1^	0.94	0.25	1.12	0.94	0.76	1.18	NA
Temperature (°C)	°C	22.34	17.10	31.10	21.46	14.30	30.40	NA
Nitrate–nitrogen (NO_3_ as N)	mg L^−1^	33.63	0.00	42.49	31.85	0.00	60.76	NA
Phosphate (PO₄^3−^)	mg L^−1^	0.00	0.00	0.00	6.80	0.00	8.75	NA
Sulfate (SO₄^2−^)	mg L^−1^	137.80	95.89	168.36	97.85	0.00	123.86	250.00 *
Copper (Cu)	mg L^−1^	0.00	0.00	0.00	0.00	0.00	0.00	0.20 *
Iron (Fe)	mg L^−1^	0.02	0.00	0.06	0.00	0.00	0.01	5.00 *
Manganese (Mn)	mg L^−1^	0.78	0.36	1.25	0.60	0.38	0.72	0.20 *
Molybdenum (Mo)	mg L^−1^	0.005	0.001	0.015	0.015	0.004	0.100	NA
Nickel (Ni)	mg L^−1^	0.00	0.00	0.00	0.00	0.00	0.00	0.20 *
Zinc (Zn)	mg L^−1^	0.00	0.00	0.00	0.00	0.00	0.00	2.00 *
K	mg L^−1^	0.16	0.06	0.24	0.24	0.22	0.26	0–2 **
Ca	meq L^−1^	4.06	2.16	5.10	4.93	4.93	5.73	0–20 **
Mg	meq L^−1^	0.78	0.36	1.25	0.60	0.38	0.72	0–5 **

Quality standard of water for agriculture as indicated by the * Mexican Federal Law [26] and ** FAO guideline for the quality of water used for irrigation [27].

**Table 3:** Nitrogen, phosphorus, potassium, and magnesium content in barley plants (*Hordeum vulgare*) irrigated with groundwater (GW) and treated wastewater (TWW).

Water Source	Nitrogen (N) mg kg^−1^	Phosphorus (P) mg kg^−1^	Potassium (K) mg kg^−1^	Magnesium (Mg) mg kg^−1^
	Grains			
GW	21,300 a	3508 a	7500 a	1600 a
TWW	11,450 b	2800 a	6300 b	1400 a
	Leaves			
GW	11,320 a	833 a	8300 b	2400 a
TWW	11,020 b	933 a	9200 a	2000 b
	Stems			
GW	7500 a	458 b	8200 a	1500 a
TWW	7100 a	691 a	8700 a	1200 b
	Roots			
GW	5500 a	933 b	8300 a	2600 a
TWW	5200 a	1566 a	8400 a	2000 b

Different letters indicate a significant difference (*p* < 0.05).

## Data Availability

Data are available upon request, but if used in another manuscript, the authors wish to be included as co-authors.
